# The Inevitable Complement—The Care and After-Care of Consumptives

**Published:** 1915-06

**Authors:** 


**The Inevitable Complement—the Care and After-care of Consumptives.** By Harold Vallow, M.D. Pp. viii., 56. London: John Bale, Sons & Danielsson Ltd. 1915.

The chief Clinical Tuberculosis Officer on the Insurance Committee of Bradford gives the results of his experiences gained at a Sanatorium and Dispensary in a large industrial area. He enforces the view that it is necessary to follow up the patients after leaving the institutions in order that the good results shall become permanent, and he writes with the object of pointing out what he considers the best methods for keeping these patients well. The booklet is one to be studied by all tuberculosis officers and those who are joining in the fight against consumption. This must have the co-operation of all political parties and philanthropists, and the author appeals to all members of the community to insist that the Government shall provide for the people what they have promised under the sanatorium provisions of the National Insurance Act.

